# Genome-Wide Identification, Evolution and Expression Analysis of the Glutathione S-Transferase Supergene Family in Euphorbiaceae

**DOI:** 10.3389/fpls.2022.808279

**Published:** 2022-03-10

**Authors:** Qiang Duan, Guo-Rui Li, Yi-Peng Qu, Dong-Xue Yin, Chun-Ling Zhang, Yong-Sheng Chen

**Affiliations:** ^1^College of Life Sciences and Food Engineering, Inner Mongolia Minzu University, Tongliao, China; ^2^Key Laboratory of Castor Breeding of the State Ethnic Affairs Commission, Tongliao, China; ^3^Inner Mongolia Industrial Engineering Research Center of Universities for Castor, Tongliao, China; ^4^Inner Mongolia Key Laboratory of Castor Breeding, Tongliao, China; ^5^Inner Mongolia Collaborative Innovation Center for Castor Industry, Tongliao, China; ^6^Inner Mongolia Engineering Research Center of Industrial Technology Innovation of Castor, Tongliao, China; ^7^Changchun Institute of Optics, Fine Mechanics and Physics, Chinese Academy of Sciences, Changchun, China

**Keywords:** Euphorbiaceae, GST, phylogeny, evolutionary pressure, expression pattern, protein interaction

## Abstract

Euphorbiaceae, a family of plants mainly grown in the tropics and subtropics, is also widely distributed all over the world and is well known for being rich in rubber, oil, medicinal materials, starch, wood and other economically important plant products. Glutathione S-transferases (GSTs) constitute a family of proteins encoded by a large supergene family and are widely expressed in animals, bacteria, fungi and plants, but with few reports of them in Euphorbiaceae plants. These proteins participate in and regulate the detoxification and oxidative stress response of heterogeneous organisms, resistance to stress, growth and development, signal transduction and other related processes. In this study, we identified and analyzed the whole genomes of four species of Euphorbiaceae, namely *Ricinus communis, Jatropha curcas, Hevea brasiliensis*, and *Manihot esculenta*, which have high economic and practical value. A total of 244 GST genes were identified. Based on their sequence characteristics and conserved domain types, the GST supergene family in Euphorbiaceae was classified into 10 subfamilies. The GST supergene families of Euphorbiaceae and Arabidopsis have been found to be highly conserved in evolution, and tandem repeats and translocations in these genes have made the greatest contributions to gene amplification here and have experienced strong purification selection. An evolutionary analysis showed that Euphorbiaceae GST genes have also evolved into new subtribes (GSTO, EF1BG, MAPEG), which may play a specific role in Euphorbiaceae. An analysis of expression patterns of the GST supergene family in Euphorbiaceae revealed the functions of these GSTs in different tissues, including resistance to stress and participation in herbicide detoxification. In addition, an interaction analysis was performed to determine the GST gene regulatory mechanism. The results of this study have laid a foundation for further analysis of the functions of the GST supergene family in Euphorbiaceae, especially in stress and herbicide detoxification. The results have also provided new ideas for the study of the regulatory mechanism of the GST supergene family, and have provided a reference for follow-up genetics and breeding work.

## Introduction

Glutathione S-transferases (GSTs) constitute a family of proteins encoded by a large supergene family widely expressed in plants, animals, bacteria and fungi, and carry out a variety of physiological functions (Wang et al., 2020). Booth et al. (1961) were the first to find glutathione S-transferase activity in an animal, and did so in rat liver slices. Shimabukuro et al. (1970) discovered glutathione S-transferase in plants for the first time while studying the detoxification mechanism for corn herbicides. With the continuous advances in sequencing technology, increasingly in-depth studies of GST genes have been carried out. In plants, glutathione S-transferase participates in a wide range of catalytic and regulatory functional networks involved in detoxification and oxidative stress responses of heterogeneous organisms, including resistance to stress, growth and development, and signal transduction. Dixon et al. (1998) focused on the function of GST, and their studies indicated that glutathione and its dependent enzymes play a central role in detoxifying exotic or endogenous active low-molecular-weight organic compounds. Kampranis et al. (2000) carried out yeast gene screening to isolate a new GST and found that the enzyme lessened the lethality of the mammalian inducer of apoptosis BAX in yeast. Wu et al. (1999) reported that the herbicide carbendazim can increase the activity of glutathione transferase by inducing the expression of the glutathione transferase gene, thus protecting rice seedlings from alachlor. Some studies have shown that transgenic tobacco plants acquired tolerance to the diphenyl ether herbicide trifluorotrifluoxane (fluorodifen) through overexpression of *CsGSTUs* in tobacco, and transgenic tobacco showed resistance to salt and drought stress (Cicero et al., 2015). Yang et al. (2016) reported that transient overexpression of *JrGSTTau1* improves the cold tolerance of walnuts, and is related to active oxygen metabolism, cell membrane protection and regulation of enzymes and stress-related genes. In addition, GST has been reported to be involved in the transport of secondary metabolites, especially the transport and accumulation of anthocyanins. Fang et al. (2020) found that, in apples, *MdGSTF6* promoters are activated and expressed when directly bound by MdMYB1, thus allowing *MdGSTF6* products to participate in the transport of anthocyanins from the cytoplasm to vacuoles; and Gomez et al. (2011) studied cellular transport of anthocyanins in grapes by using the unique autofluorescence characteristics of anthocyanins, and proved that GST mediates the transport of anthocyanins in grapes and leads to the final accumulation of the anthocyanins in vacuoles.

There are extreme sequence differences between different subfamilies of the GST supergene family, but the overall structure of the GST protein is highly conserved. Soluble GST is usually a hydrophilic protein formed as a result of the dimerization of its approximately 26 kDa subunits. The ability of GST to form homodimers and heterodimers directly determines the diversity of GST in plants (Edwards et al., 2000). The typical GST protein domain contains two active sites: the highly conserved N-terminal domain glutathione (GSH) binding site (G-site), which is used to specifically accept GSH, and which can promote formation of catalytic glutathione S negative ions; and the complex C-terminal cosubstrate binding domain site (H-site), which provides a highly conserved aspartic acid residue and most of the residues that can bind to hydrophobic ligands for glutathione binding. Classification of the GST supergene family in plants is complicated. Lallement et al. (2014) classified plant GSTs into 14 types, namely phi (GSTF), tau (GSTU), lambda (GSTL), theta (GSTT), zeta (GSTZ), DHAR, EF1B γ, TCHQD, GHR, iota (GSTI), hemerythrin (GSTH), Ure2p, microsomal prostaglandin E-synthase 2 (mPGES-2), and metaxin. Kappa GSTs and mPGES-1 (microsomal prostaglandin E-synthetase type 1) belong to the subclass MAPEGs (membrane-associated proteins in the metabolism of eicosanoids and glutathione) and are usually integrated into the GST superfamily. In addition, there are other GST subfamilies such as Omega (GSTO) that have been identified in plants (Dixon et al., 2002; Bresell et al., 2005).

At present, thanks to continuous progress of sequencing technology and reduction of sequencing costs, increasing numbers of GST genes have been identified in non-model plants, including 53 *Arabidopsis thaliana*, 62 *Pyrus* spp., 52 *Malus pumila* Mill., 70 *Gossypium hirsutum* L., 49 *Cucumis melo* L., 90 *Lycopersicon esculentum* Miller, 79 *Oryza sativa* L., and 85 *Capsicum annuum* L. genes (Sappl et al., 2009; Jain et al., 2010; Islam et al., 2017, 2019; Xu et al., 2017; Wang et al., 2018, 2020; Fang et al., 2020). However, there are few studies on the GST supergene family in Euphorbiaceae, despite the diversity and importance of these plants. Liao et al. (2018) identified the *Manihot esculenta* GST supergene family, but did not analyze it at the chromosome level or study its gene evolution. To date, 8,910 species belonging to 322 genera of Euphorbiaceae, including trees, shrubs and herbs, have been identified and found widely distributed all around the world, but mainly in tropical and subtropical regions. Euphorbiaceae plants are well known for their economically important products such as rubber, oil, medicine, starch, and wood. In this regard, *Ricinus communis*, *Jatropha curcas*, *Hevea brasiliensis*, and *Manihot esculenta* are four important representative examples of Euphorbiaceae. The oil content of the seeds of *R. communis* is 2–3 times that of soybeans; *R. communis* oil is widely used in chemical, aerospace, pharmaceutical and other industries, and hence this plant is an important oil crop worldwide. *J. curcas* is a high-quality renewable-energy plant whose seeds have an oil content of up to 40% and good fluidity; this oil shows good blending with diesel, gasoline and alcohol, and can be directly used to produce biodiesel. *M. esculenta* is one of the three most important root and tuber crops in the world, displaying an extraordinarily high rate of utilization of light, heat and water resources; it is an important source of food throughout the world, with a biomass energy yield per unit area higher than that of almost all other cultivated crops—and its storage root is rich in carbohydrates, the main raw material for the production of fuel alcohol. *H. brasiliensis* is the only rubber-producing crop widely cultivated and commercially applied at present; note in this regard that natural rubber is a scarce resource of strategic significance, being widely used in various fields including those involved in national defense and national economic production.

In the current work, *R. communis*, *J. curcas*, *H. brasiliensis*, and *M. esculenta*, were selected for having their GST supergene families identified. On this basis, their gene structure, biochemical characteristics, evolutionary relationship, expression patterns and protein interactions were analyzed. A total of 244 GST genes were identified. Based on their sequence characteristics and conserved domain types, the GST supergene family in Euphorbiaceae was classified into 10 subfamilies. Collinearity analysis and evolutionary selection stress analysis revealed the amplification and evolution of the GST gene in Euphorbiaceae. In addition, the expression response pattern of the GST supergene family was analyzed according to the expression profile of this supergene family in herbicide detoxification, resistance to stress, and different tissues. Therefore, the results of this study have laid a foundation for the analysis of the functions of the GST supergene family in Euphorbiaceae, and have provided a reference for related genetics and breeding work.

## Results

### Identification of Members of the Glutathione S-Transferase Supergene Family in Euphorbiaceae and Analysis of Their Protein Physicochemical Properties

In our current study, the *M. esculenta* GST supergene family was re-analyzed, and the genome of this species and the genomes of three other representative Euphorbiaceae species, namely *Ricinus communis*, *Jatropha curcas*, and *Hevea brasiliensis*, were compared. As shown in Table 1, for these four representative species of Euphorbiaceae, the number of members of the GST supergene family was found to be positively correlated with their genome size; here the Brazilian *H. brasiliensis* and *M. esculenta* species were found to have both the largest genomes and most members. In order to determine the number of members of the GST supergene family in Euphorbiaceae, local BLASTP and HMMER comparisons were carried out. A total of 244 members of the GST supergene family were identified, including 44 *R. communis*, 38 *J. curcas*, 85 *H. brasiliensis* and 77 *M. esculenta* gene members, which were named according to species, chromosome number, subfamily type and gene position on chromosome.

**TABLE 1:** The GST supergene family in Euphorbiaceae.

Family	Species	Gene number	Assembly version	Genome size (Mb)
Brassicaceae Burnett	*Arabidopsis thaliana*	53	TAIR10.1	115
Euphorbiaceae	*Ricinus communis*	44	Rc039	324
	*Jatropha curcas*	38	RJC1_Hi-C	257
	*Hevea brasiliensis*	85	ASM165405 v1	1,290
	*Manihot esculenta*	77	Manihot esculenta v8.0	620

Members of the GST supergene family in Euphorbiaceae were systematically evaluated, including for gene and protein lengths, protein molecular weight, isoelectric point, subcellular localization (see Supplementary Tables 1–4). The results showed little difference between the number of residues and relative molecular weight of most GST family proteins of Euphorbiaceae, but significant differences between several members, with only 162 residues and the lowest relative molecular weight of 18,473.86 for JcGSTT2, but 1,531 residues and the highest relative molecular weight of 174,129.27 for RcGSTU15. A wide range of isoelectric points was found, from the lowest of 4.78 for RcGSTU8 to the highest of 9.61 for JcGSTT3. The grand average of hydropathy was calculated to be negative in all cases except for RcGSTU8, MeGSTU3, JcGSTU11, RcGSTL2, and RcGSTU7; hence, most of the GST family proteins of Euphorbiaceae were shown to be hydrophilic. The results of subcellular localization analysis showed proteins of the Euphorbiaceae GST supergene family to be widely distributed, namely in the cytoplasm, plasma membrane, mitochondrion, chloroplast, periplasm, adventitia and extracellular regions. Nevertheless, most of the proteins of this supergene family were found to be located in the cytoplasm.

### Analysis of Glutathione S-Transferase Gene Structure and Conserved Protein Motifs of Euphorbiaceae

**FIGURE 1:**
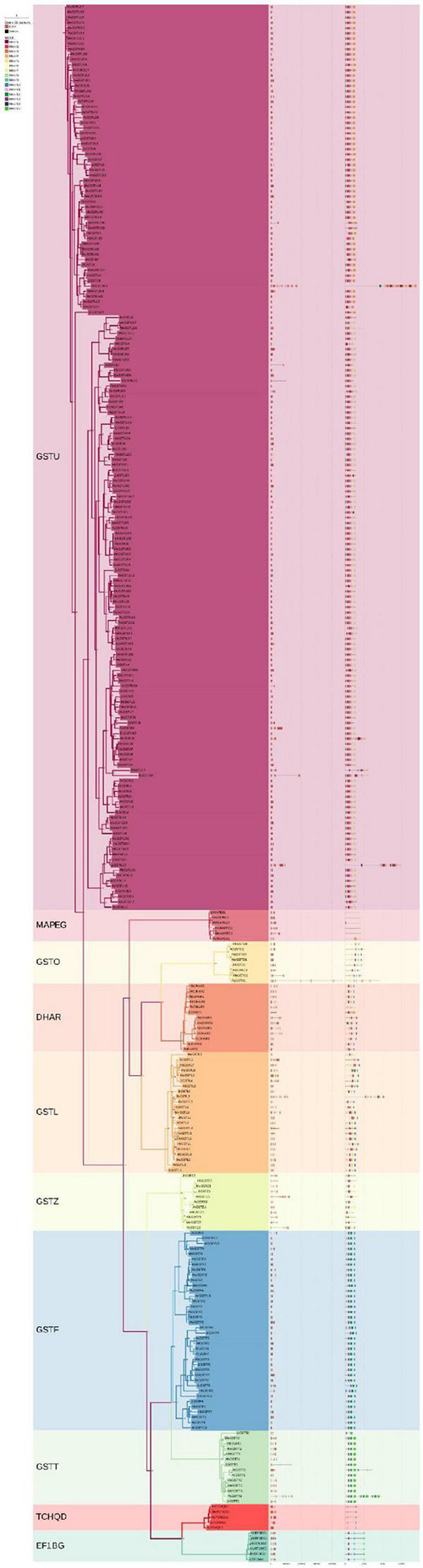
Classification, gene structure and conserved domains of the GST supergene families in Euphorbiaceae and *Arabidopsis thaliana.*

**FIGURE 2:**
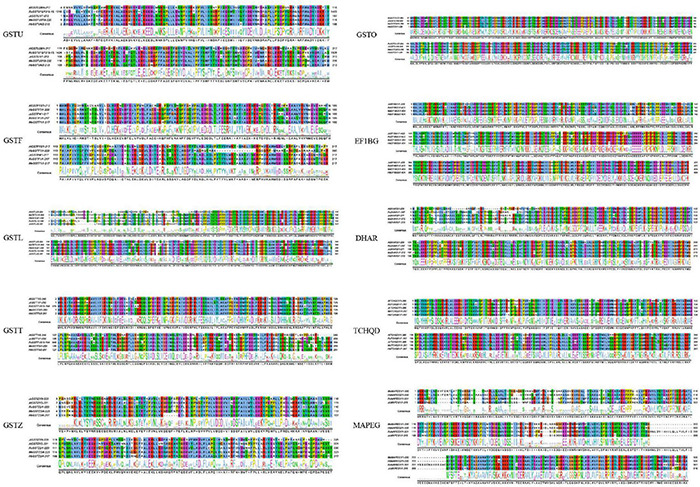
Sequence characteristics of the GST supergene family in Euphorbiaceae. The levels of conservation of the residues in the protein sequences are illustrated using the ClustalX color scheme.

To further investigate the gene and protein structures of the GST supergene family in Euphorbiaceae, gene structure was analyzed based on the genome annotation files of various species and by using the written script, with this analysis including 244 GST genes of Euphorbiaceae and 53 GST genes of Arabidopsis. The distribution of exons and introns of the GST genes in Euphorbiaceae was visualized. Between 1 and 29 exons and between 0 and 30 introns were identified (Figure 1). “Motif” refers to the conserved sequence of DNA, protein and other biological macromolecules, and is another structural level between the secondary and tertiary structures. The results of an MEME analysis showed a total of 15 such motifs for the members of the GST supergene family in Euphorbiaceae, with between 1 and 10 such motifs for any single member (Figure 1). The motif structures differed considerably between members of the different subfamilies, but not much at all between members of the same subfamily (Figure 2). The analyses here showed most members of the subclan DHAR having Motif1, Motif5, Motif6, Motif8, and Motif10, most members of subclan EF1BG having Motif6, Motif8, Motif9, Motif10, and Motif13, most members of subclan GSTF having Motif5, Motif6, Motif9, Motif10, Motif11, Motif12, and Motif13, most members of subclan GSTL having Motif1, Motif5, Motif6, and Motif10, most members of subclan GSTO having Motif1, Motif5, Motif6, Motif8, and Motif10, and most members of subclan GSTT having Motif6, Motif11, Motif12, Motif13, and Motif13.

The analyses also showed most members of subfamily GSTU having Motif1, Motif2, Motif3, Motif5, Motif6, Motif7, Motif8, Motif9, and Motif14, most members of subfamily GSTZ having Motif1, Motif5, Motif6, Motif7, Motif9, and Motif14, most members of subfamily MAPEG having Motif6, and all members of subfamily TCHQD1 having Motif5, Motif6, Motif10 and Motif11. While the structure of the GST supergene family in Euphorbiaceae was found to be relatively complex, the member sequences of each subfamily were highly conserved, perhaps suggesting these subfamilies to be functionally differentiated and participating in the regulation of different functions. It was confirmed that Motif1, Motif3 and Motif11 corresponded to thioredoxin (TRX), that Motif2, Motif4, Mitif6, Motif7, Motif10, Motif12, and Motif15 corresponded to GSTC, and that Motif5 and Motif13 corresponded to GSTN.

**FIGURE 3:**
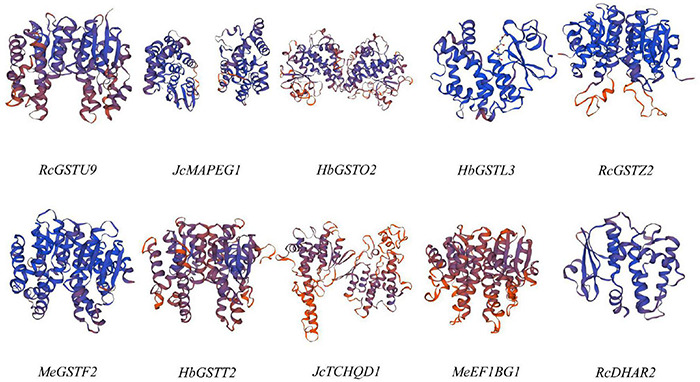
Three-dimensional structural models of the Euphorbiaceae GST supergene family proteins. The color gradient shows the GMQE confidence, with red indicating lowest confidence and blue highest confidence.

In order to further investigate the structure of GST proteins of Euphorbiaceae, the representative GST subfamily proteins of Euphorbiaceae were modeled using SWISS-MODEL (Waterhouse et al., 2018). Despite the considerable sequence differences between the different subfamilies of the Euphorbiaceae GST supergene family (Figure 2), the three-dimensional structures of the GST supergene family were all found to be symmetrical dimers and were similar to one another (Figure 3).

### Analysis of *Cis*-Acting Elements of the Glutathione S-Transferase Gene of Euphorbiaceae

In order to explore the potential function and regulatory model of the GST supergene family of Euphorbiaceae, the *cis*-acting elements in the promoter region of 244 members were predicted. The results showed that all GST genes had light response elements (Box4, GT1-motif, G-box, GA-motif, TCT-motif, ATCT-motif, etc.). At the same time, auxin and salicylic acid response elements were also numerous, indicating that GST genes in Euphorbiaceae may be regulated by light, hormones and other factors. Various stress response elements such as defense and stress element (TC-rich repeats), drought-inducer element (MBS), low-temperature response element (LTR), hypoxia inducible element (GC-motif) and anaerobic inducible element (ARE) were also detected, which is consistent with the function of the GST gene involved in stress regulation. In addition, *cis*-acting elements related to development were found in the promoter region of some Euphorbiaceae GST genes, such as circadian rhythm control element (Circadian), palisade mesophyll cell differentiation element (HD-Zip1), Zein metabolic regulatory element (O2-site), among others, indicating that these GST genes may be involved in regulation of growth and development of Euphorbiaceae plants (Supplementary Figures 1–4).

### Chromosome Mapping and Replication Analysis of the Glutathione S-Transferase Supergene Family in Euphorbiaceae

**FIGURE 4:**
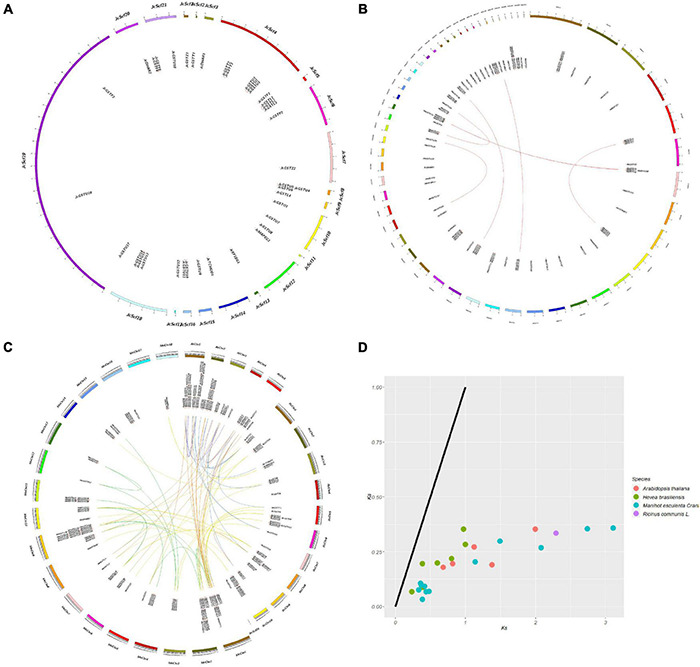
Chromosome mappings, collinearity analyses, and evolutionary selection pressure analyses of GST genes in Euphorbiaceae. Collinearity analysis of *Jatropha curcas* **(A)**. Collinearity analysis of *Hevea brasiliensis* **(B)**. Collinearity analyses of *Ricinus communis, Manihot esculenta* and *Arabidopsis thaliana* **(C)**. The purple lines represent the collinear relationship within the *A. thaliana* species, the red lines represent the collinear relationship within the *R. communis* species, the green lines represent the collinear relationship within the *M. esculenta* species, the yellow lines represent the collinear relationship between *R. communis* and *M. esculenta* species, the blue lines represent the collinear relationship between *R. communis* and *A. thaliana* species, and the orange lines represent the collinear relationship between *M. esculenta* and *A. thaliana* species. Evolutionary selection pressure of Euphorbiaceae **(D)**.

The distribution of genes on plant chromosomes is closely related to participation of the chromosomes in gene expression and their importance to plant growth and development. In order to investigate the replication pattern and evolutionary mechanism of Euphorbiaceae GST genes, chromosome mapping analyses (Figures 4A–C) were carried out on the four studied representative species of Euphorbiaceae.

Because the reference genome of *J. curcas* has not been assembled at the chromosome level, we mapped and analyzed it at the Scafford level (Figure 4A). In addition, the reference genomes of different varieties of *H. brasiliensis* were assembled at different levels (Tang et al., 2016; Liu et al., 2020), so we analyzed them at the Scafford and chromosome levels (Figure 4B and Supplementary Figure 5).

In order to investigate the evolutionary relationship between members of the GST supergene family in Euphorbiaceae, we carried out collinear and evolutionary selection pressure analyses (Figure 4D) on the four studied representative species of Euphorbiaceae and on *Arabidopsis thaliana*. The non-synonymous substitution rate (Ka), synonymous substitution rate (Ks) and their ratio (Ka/Ks) for fragment repeat gene pairs were calculated to investigate their roles in the amplification of GST genes in Euphorbiaceae. The Ka values of all gene pairs were less than Ks, and all Ka/Ks values were less than 0.6. This set of results suggested that the GST supergene family in Euphorbiaceae has experienced selection for strong purification during evolution to reduce harmful mutations after fragment duplication (Figure 4D and Supplementary Table 5).

A collinear relationship between Euphorbiaceae and *A. thaliana* was confirmed. Homologous gene pairs were identified in four of the species studied, including 9 pairs in *A. thaliana*, 1 pair in *R. communis*, 7 pairs in *H. brasiliensis* and 12 pairs in *M. esculenta* (Supplementary Table 5). Note that we did not find any homologous gene pairs in *J. curcas*, perhaps due to the imperfections in the assembly of the reference genome of this species. In addition, we analyzed the replication events of the GST supergene family in Euphorbiaceae. The replication events of this supergene family were found to be relatively complex; here, tandem repeats and translocations were indicated to have made the greatest contributions to the gene amplification of the Euphorbiaceae GST supergene family, followed by whole genome replication and fragment replication, with only a small number of members showing tandem replication and insertion of some other genes. Similar gene replication events were also indicated for the Arabidopsis GST supergene family. Of the 53 members of the Arabidopsis GST supergene family, 27 of them resulted from tandem repeats. Some members of the GST supergene family were found to be arranged very closely to each other on the chromosomes of Euphorbiaceae and *A. thaliana*, and into clusters, indicating that large-scale fragment replication events may have occurred in the course of evolution.

### Analysis of the Evolution and Development of the Glutathione S-Transferase Supergene Family

**FIGURE 5:**
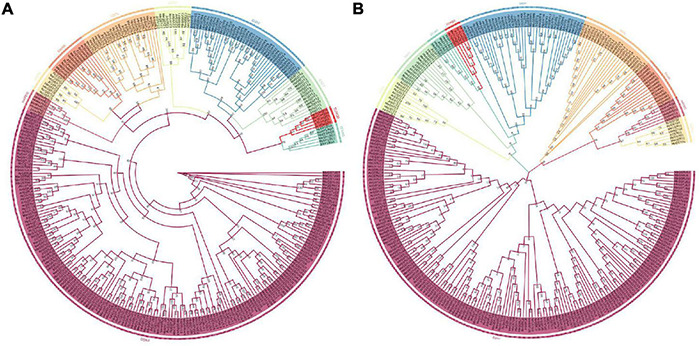
Adjacent phylogenetic tree and maximum likelihood phylogenetic tree of the Euphorbiaceae and Arabidopsis GST supergene families. Evolutionary development tree of the Euphorbiaceae and *Arabidopsis thaliana* GST supergene families resulting from application of the adjacency method **(A)**. Evolutionary development tree of the Euphorbiaceae and *A. thaliana* GST supergene families resulting from application of the maximum likelihood method **(B)**.

In order to investigate the evolution and development of the GST supergene family in Euphorbiaceae, the respective phylogenetic trees of Euphorbiaceae and *A. thaliana* (Figures 1, 5) were constructed. In order to ensure the accuracy of the results, the adjacency and maximum likelihood methods were both deployed, with the LG + G model used for the maximum likelihood method. The results showed the presence of 244 GST gene members of Euphorbiaceae and 53 GST members of *A. thaliana*. The 297 genes were divided into 10 subfamilies: GSTU, GSTO, GSTZ, GSTL, GSTF, GSTT, MAPEG, DHAR, EF1BG, and TCHQD. The motif structure of the MAPEG and TCHQD subfamilies was found to be relatively simple, and the respective motif structures of the members of each subfamily were found to be highly consistent (Figure 1). Subfamilies GSTO, EF1BG and MAPEG were not found in the Arabidopsis GST supergene family, but all of the other seven subfamilies were found in both the Euphorbiaceae and Arabidopsis GST supergene families (Figures 1, 5), perhaps indicative of the similar evolutions of Euphorbiaceae and Arabidopsis. Interestingly, the analyses showed GSTO, EF1BG and MAPEG to be the last three subfamilies (Figure 1) in the evolution of the GST supergene family, and these three subfamilies may play a specific role in Euphorbiaceae.

### Analysis of the Expression Patterns of the Glutathione S-Transferase Supergene Family in Euphorbiaceae

**FIGURE 6:**
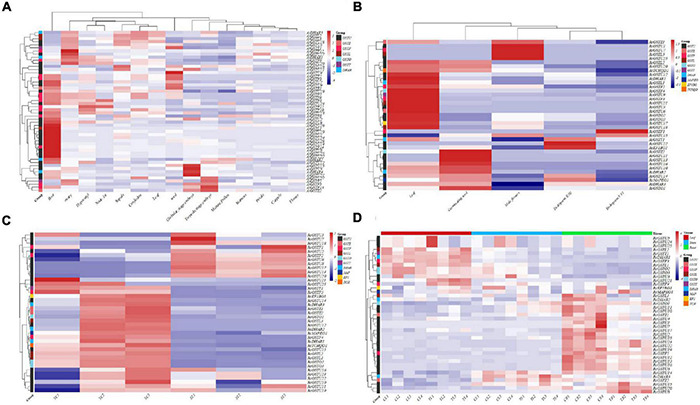
Analysis of gene expression profiles of Euphorbiaceae and *Arabidopsis thaliana.* Heat maps of expression profiles of the indicated genes in various tissues of *A. thaliana* **(A)**, various tissues of *Ricinus communis* **(B)**, *R. communis* infected with *Sclerotinia sclerotiorum* **(C)**, and *R. communis* treated with herbicide **(D)**.

Because the analysis of structure and evolution showed that members of the GST supergene family of Euphorbiaceae are highly similar, the expression patterns and functions of GST genes in Euphorbiaceae were studied, with R. communis of Euphorbiaceae as the representative organism. We obtained the relevant Euphorbiaceae expression data from the NCBI GEO database, including expression in II/III endosperm, V/VI endosperm, germinated seeds, leaves and male flowers, to investigate the expression patterns of GST genes in different tissues (Brown et al., 2012). In addition, we obtained the expression data for different tissues of *A. thaliana* to compare the expression patterns of the Euphorbiaceae and Arabidopsis GST supergene families (Winter et al., 2007). The data for plant leaves infected with *Sclerotinia sclerotiorum* were obtained from the NCBI GEO database to investigate the patterns of responses of Euphorbiaceae GST genes to biological stress (Sucher et al., 2020). In order to investigate the functions of Euphorbiaceae GST genes in response to abiotic stress, especially in the detoxification of herbicides, we randomly selected *R. communis* seedlings at the 3-leaf growth stage as experimental materials. The *R. communis* populations were sprayed with a normal concentration (6 g/mu)-25% of sulfenesulfuron as the treatment group, and the two *R. communis* populations were sprayed with the same amount of water without herbicide treatment as the control group; the roots, stems and leaves were all sprayed in each case. When drawing the expression heat map, all of the expression levels were standardized (Figure 6).

In order to better understand the patterns of GST gene expression in Euphorbiaceae, we eliminated genes showing zero expression in all samples, and then hierarchically clustered the remaining GST genes, including subfamily and tissue clustering. The results showed which GST genes of Euphorbiaceae displayed different expression patterns in different tissues, and the degree of tissue specificity (Figures 6B,D). Most GST genes in Euphorbiaceae were found to be highly expressed only in specific tissues, with most GST genes highly expressed in roots, leaves and seeds, consistent with expression of GST genes in *A. thaliana* (Figure 6A). Considerably more GST genes were found to be highly expressed in root tissues than in other tissues. At the same time, the subfamily clustering of GST genes showed no GST subfamily genes expressed at specific sites. Interestingly, we found that several GST subfamily genes (Figure 6) were often included in the highly expressed clustered modules in specific tissues, which may indicate a possible co-regulation of GST genes in specific tissues.

Measurements of biological stress of Euphorbiaceae plants infected with *Sclerotinia sclerotiorum* showed that some GSTU and GSTF genes were up-regulated after the exposure to the stress, with these up-regulated genes including *RcGSTU2*, *RcGSTU3*, *RcGSTU6*, *RcGSTU7*, *RcGSTU8*, *RcGSTU13*, *RcGSTU17*, *RcGSTU18*, *RcGSTU20*, *RcGSTF1* and *RcGSTF2* (Figure 6C). The log2FC (fold change) values of *RcGSTU13*, *RcGSTU8* and *RcGSTU20* were, respectively, 8.05, 7.34, and 4.10, and the average expression level of *RcGSTU13* was as high as 22,731.62.

A heat map was produced showing the response of Euphorbiaceae plants to abiotic stress in roots, stems and leaves after being sprayed with the herbicide rimsulfuron (Figure 6D). In leaves, expression of *RcGSTU13*, *RcGSTU4*, *RcGSTU21*, *RcGSTU20*, *RcGSTU23*, *RcGSTU22*, *RcGSTU8*, *RcGSTU17*, *RcGSTU11*, *RcGSTU10*, and *RcGSTF4* were all up-regulated, and *RcGSTU13*, *RcGSTU4*, and *RcGSTU21* were specifically up-regulated 41-, 7-, and 4-fold, respectively. In stems, expression of *RcGSTU23*, *RcGSTU22*, *RcGSTU13*, *RcGSTU8*, *RcGSTU20*, *RcGSTU4*, *RcGSTU16*, *RcGSTU6*, *RcGSTU11*, and *RcGSTU10* were all up-regulated, and *RcGSTU23* and *RcGSTU22* were each up-regulated 13-fold, and *RcGSTU13* and *RcGSTU8* were both up-regulated 7-fold. In roots, expression of *RcGSTU8*, *RcGSTU3*, *RcGSTU21*, *RcGSTU15* and *RcGSTU20* were all up-regulated, and *RcGSTU8* and *RcGSTU3* were up-regulated 32- and 6-fold, respectively. Note that *RcGSTU8* was differentially expressed in rhizome and leaf tissues after herbicide treatment, and up-regulated significantly. The overall expression trend of these genes obtained by QPCR analysis was basically consistent with that of RNA-seq analysis (Supplementary Table 6 and Figure 6).

### Analysis of Protein Interactions Within the Glutathione S-Transferase Supergene Family in Euphorbiaceae

**FIGURE 7:**
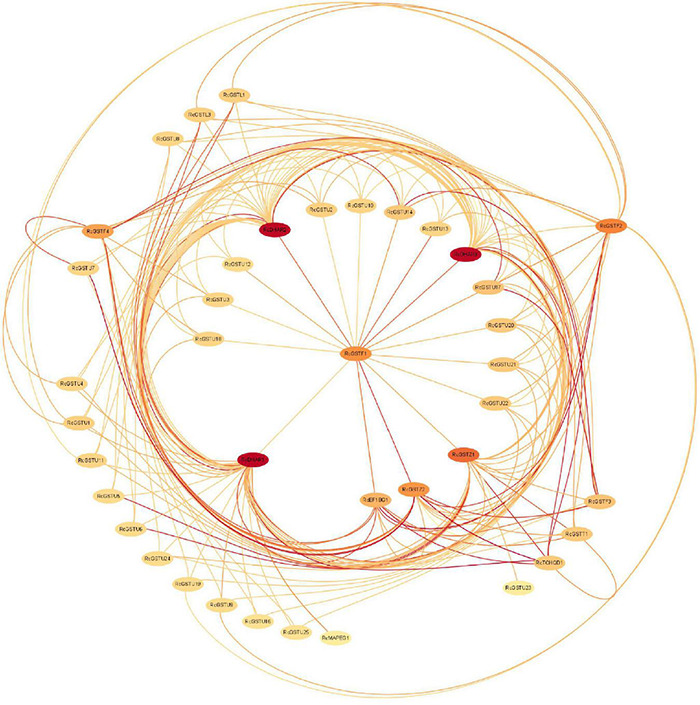
GST supergene family protein interaction network for Euphorbiaceae. Darker colors indicate higher Node Degrees.

In order to investigate whether co-regulation occurs in the GST supergene family in Euphorbiaceae, we carried out a protein-interaction analysis (Figure 7). The results provided further evidence for our previous idea suggesting RcGSTF1 to be at the core of the interaction network, with the three members of the subfamily DHAR, namely RcDHAR1, RcDHAR2, and RcDHAR3, being the most obvious core nodes. In addition, RcGSTZ1, RcGSTZ2, RcGSTF1, RcGSTF2, RcGSTF4, and RcEF1BG1 were indicated by the current analysis to all play an important role in the interaction network. Some members of the non-GSTU subfamily were seen to occupy HUB status in the network and to interact with multiple GSTU proteins, which may imply internal regulation of GST supergene family proteins in Euphorbiaceae.

## Discussion

Glutathione S-transferases display a variety of biological functions, including detoxification and oxidative stress, resistance to stress, and signal transduction (Banki et al., 1996; Smith et al., 2003; Gao et al., 2020). However, few studies had been conducted on the GST supergene family in Euphorbiaceae, and hence the functions of these proteins in this family of plants are poorly understood. Euphorbiaceae is rich in rubber, oil, medicinal materials, starch, wood and other economically important plant products and scarce resources. The Euphorbiaceae species *Ricinus communis*, *Jatropha curcas*, *Hevea brasiliensis*, and *Manihot esculenta* are important economic crops or food sources around the world. Euphorbiaceae plants and plant products are widely used in industry, aerospace, food processing, medicine and other industries, with high economic and practical value. In this study, the GST supergene family in Euphorbiaceae was identified at the whole genome level, and on this basis, its gene structure, biochemical characteristics, evolutionary relationships, expression patterns and protein interactions were analyzed. The purpose of this work was to provide new ideas and references for related follow-up research and genetics and breeding work.

The results of our gene structure, conserved motif and three-dimensional structure analyses verified our proposed classification of the GST supergene family in Euphorbiaceae (Figures 1–3). The members of each subfamily were shown to be highly conserved in evolution. The seven subfamilies other than MAPEG, GSTO, and EF1BG were found to be common to Euphorbiaceae plants and the model plant *Arabidopsis thaliana*. This result provided a powerful reference for predicting the function of the GST supergene family in Euphorbiaceae (Figures 1, 5). Interestingly, the resulting interspecific phylogenetic tree showed that the MAPEG, GSTO, and EF1BG subfamilies appeared last during evolution of GST, and this observation suggested that their members may play a special role in Euphorbiaceae. There are few studies on MAPEG in plants, and its function is more complex than those of other GST subfamilies. Most MAPEG proteins are mainly involved in the synthesis of eicosanes, leukotrienes and prostaglandins, while human MAPEG proteins show more typical GST catalytic activity. In addition, it has been shown that MGST1 also catalyzes the reduction of GSH-dependent lipid hydroperoxides (Morgenstern and DePierre, 1983; Mosialou et al., 1995). MGST1 is considered to be primarily a detoxifying enzyme involved in cellular defense against toxic foreign substances and metabolites produced by oxidative stress. We found that expression of *RcMAPEG1* in the root tissue of Euphorbiaceae was up-regulated after herbicide spraying, which suggested that it may be involved in herbicide detoxification in the root tissue of Euphorbiaceae. EF1BG is the coding protein of the eukaryotic translation elongation factor γ subunit. Hiraga et al. (1993) used gene blocking experiments to show that EF1Bβ is necessary for growth of *Saccharomyces cerevisiae*. Olarewaju et al. (2004) found that deletion of either of the two genes encoding eEF1Bγ will increase *Saccharomyces cerevisiae* resistance to oxidative stress. In our study, *RcEF1BG1* was significantly down-regulated in the leaf tissues of Euphorbiaceae infected by *Sclerotinia sclerotiorum*, which was consistent with studies using *Saccharomyces cerevisiae*. We speculated that *RcEF1BG1* is involved in the response of Euphorbiaceae to biological stress. The function of GSTO is different from that typical of GSTs. It has been found that GSTO1-1 has the same mercaptan transferase and glutathione-dependent dehydroascorbate reductase activity as glutathione reductase (Zakharyan et al., 2001). In addition, the GSTO coding gene can significantly improve the tolerance of *Escherichia coli* AW3110 (DE3) to arsenite, and plays an important role in arsenic detoxification of *Ruditapes philippinarum* (Chen et al., 2018). We found that expression of *RcGSTO1* was up-regulated in the stem tissue of Euphorbiaceae treated with herbicide, suggesting that *RcGSTO1* may play a role in abiotic stress resistance in the stem tissue of Euphorbiaceae.

We obtained expression profile data for various Euphorbiaceae tissues, including endosperms at the II/III stage, endosperms at the V/VI stage, germinated seeds, leaves, male flowers, roots and stem segments, as well as 15 *A. thaliana* tissues: cotyledons, hypocotyls, first stem nodes, sepals, petals, stamens, carpels, mature pollen, seeds, leaves, flowers, roots, globular embryos, torpedo embryos, and ovaries. The purpose of this study was to investigate the functions of the GST supergene family in different tissues and different developmental stages of Euphorbiaceae. The GST genes of Euphorbiaceae showed significant tissue specificity, and the expression of GST genes maintained high levels in roots, leaves and seeds, consistent with the expression of GST genes in *A. thaliana*. Note that considerably more GST genes were shown to be highly expressed in root tissue than in other tissues. Out of the 37 GSTUs in sorghum, 19 were previously reported to be expressed in the root (Chi et al., 2011), suggesting an important role for GST in plant root tissue. AtGSTF8 and AtGSTU19 enzymes were reported by Horváth et al. (2019) to be involved in maintaining redox homeostasis in the Arabidopsis root meristem region. The expression of *AtGSTU* in *A. thaliana* has been shown in some studies to lead to a high level of glutathione expression, promote the growth of roots and buds, and improve the ability of *A. thaliana* to resist stress (Kao et al., 2016). In Euphorbiaceae, *RcGSTF1* and *RcGSTU6*, *RcGSTU12*, *RcGSTU13*, *RcGSTU16*, *RcGSTU19*, *RcGSTU20*, *RcGSTU22*, and *RcGSTU23* are also highly expressed in root tissue, even after abiotic stress, consistent with *A. thaliana* being involved in maintaining redox homeostasis in Euphorbiaceae root tissue. In a study of the response of *H. brasiliensis* to ethylene, Nakano et al. (2021) found that expression of members of the GSTU subfamily was highly induced by ethephon treatment to cope with abiotic stress, and also reflected the conservation of the function of the GST supergene family in Euphorbiaceae plants. Glutathione, paraquat, copper and naphthylacetic acid (NAA) were demonstrated by Smith et al. (2003) to induce expression of *AtGSTF2* in the roots of *A. thaliana* seedlings independently of the ethylene sensing mechanism, and participate in auxin-related regulation and transport. In Euphorbiaceae, *RcGSTF1* always maintained a high level of expression in root tissue, while *RcGSTF3* maintained a high level in leaf tissue. *RcGSTF2* was significantly up-regulated in leaves infected by *Sclerotinia sclerotiorum*, and *RcGSTF4* was significantly up-regulated in leaves treated with herbicides, which further confirmed our previous speculation. Different GST members belonging to the same subfamily may play different functions in different tissues. *RcGSTF1* and *RcGSTF3* may be involved in regulating growth and development of root tissues and leaves of Euphorbiaceae, while *RcGSTF2* and *RcGSTF4* may be involved in regulating the stress response of Euphorbiaceae. *CmGSTU7*, *CmGSTU10*, *CmGSTU18*, *CmGSTF2*, and *CmGSTL1* were shown to play an important role in the stress response of *Cucumis melo* L. to autotoxicity mediated by cinnamic acid in the root system of *Cucumis melo* L. seedlings (Wang et al., 2020). This co-regulation activity among members of different subfamilies of GST is also present in Euphorbiaceae plants.

After being infected with *Sclerotinia sclerotiorum*, the expression of *RcGSTU13* was up-regulated 265-fold, suggesting an important role played by *RcGSTU13* in regulating the process by which Euphorbiaceae plants resist biological stress. Rapid detoxification of herbicides through glutathione coupling has been reported to be found in many plants such as rice, wheat, soybean, corn and other crops. The application of exogenous pesticides to such crops has been shown to induce the expression of GSTU, a protein shown to degrade the applied pesticides and hence reduce pesticide-induced damage to the crops (Thom et al., 2002; Dixon et al., 2003; Axarli et al., 2009; Yang et al., 2009; Benekos et al., 2010; Taylor et al., 2013). In *Phaseolus vulgaris*, *PvGSTU1* and *PvGSTU2* were induced by fluazifoP-buty. An activity analysis showed high levels of coupling of *PvGSTU1* and especially *PvGSTU2* to 1-chloro-2,4-dinitrobenzene (CDNB) and triazine, and to acetylanilines and nitroanisole herbicides (Chronopoulou et al., 2012). In the root tissue of Euphorbiaceae, *RcGSTU8* and *RcGSTU3* were up-regulated, respectively, 32- and 6-fold after the plants were treated with the herbicide rimsulfuron; *RcGSTU8* and *RcGSTU3* were thus suggested to be involved in the process of herbicide detoxification.

Joint regulation among members of different subfamilies of the GST supergene family in Euphorbiaceae is further confirmed by protein interaction network analysis (Figure 7). We examined the HUB status of the GSTF and DHAR subfamilies in the GST interaction network. In the interaction network, RcGSTF1, RcDHAR1, RcDHAR2, and RcDHAR3 have special roles in the GST protein interaction network. RcGST1 interacts with all the highly weighted GST members in the network, and the three DHAR subfamily members who directly interact with it are the three most important cores in the network. Interestingly, it was found that *RcDHAR1* is highly expressed in leaves, *RcDHAR2* in roots, and *RcDHAR3* in stems (Figure 6D), consistent with our previous speculation. We also speculate that *RcGSTF1* may be upstream of the entire GST regulatory network, and regulate it by mediating *RcDHAR1*, *RcDHAR2*, and *RcDHAR3.* This proposal may provide a basis for further study of the regulatory mechanism of the GST supergene family of Euphorbiaceae.

## Conclusion

Glutathione S-transferases constitute a family of proteins encoded by a large supergene family, are widely expressed in plants, animals, bacteria and fungi, and display a variety of physiological functions. In this study, we selected four species of Euphorbiaceae with high economic and practical value—*Ricinus communis*, *Jatropha curcas*, *Hevea brasiliensis* and *Manihot esculenta*—and identified 244 GST genes. According to sequence characteristics and conserved domain types, the GST supergene family in Euphorbiaceae was classified into 10 subfamilies. Collinear analysis and evolutionary selection pressure analysis showed that the GST supergene families of Euphorbiaceae and Arabidopsis were highly conserved in evolution. In the course of evolution, tandem repeats and translocations made the greatest contributions to gene amplification of the Euphorbiaceae GST supergene family, which has experienced strong purification selection. Evolutionary analysis showed that while the Euphorbiaceae and Arabidopsis GST superfamilies are highly conserved, new subfamilies (GSTO, EF1BG, MAPEG) have evolved, and may play a specific role in Euphorbiaceae.

The expression patterns of the GST supergene family in Euphorbiaceae in different tissues and under biological and abiotic stress were studied. Expression of GST family genes was found to be concentrated in the roots of the plants, indicating tissue-specific expression. Functions of GST include detoxification and resistance to oxidative and other stresses, growth and development, and signal transduction in a variety of organisms. In addition, members of the Euphorbiaceae GST supergene family are specifically expressed in different tissue locations in the form of several subfamily combinations, with protein interaction analysis confirmed our findings. The results of this study lay a foundation for further analysis of the functions of the GST supergene family in Euphorbiaceae, especially in stress and herbicide detoxification, and provide new ideas for the study of regulatory mechanisms of the GST supergene family. Moreover, this study also provides a powerful reference for investigations of the genetics and breeding of herbicide-resistant transgenic plants.

## Materials and Methods

### Materials

The seeds of *Ricinus communis* Zibi No. 5 were obtained from the Institute of Agriculture and Animal Husbandry Science, Tongliao City, Inner Mongolia, and were planted in the substrate of pastoral soil, with a ratio of vermiculite to nutritious soil of 1:2:1 in the laboratory. Randomly selected seedlings of Euphorbiaceae plants at the 3-leaf growth stage were selected as materials used for our experiments. The populations of Euphorbiaceae, which had been sprayed with a normal concentration of herbicides (6 g/mu, 25% rimsulfuron) were taken as the treatment (test) group, and the two populations of Euphorbiaceae sprayed with the same amount of water without herbicide were taken as the control group. Four plants were randomly sampled from each treatment group, the leaves, stem segments and roots of each plant were selected, and the samples were recorded as the CL group, TL group, CS group, TS group, CR group and TR group, respectively. The samples were frozen in liquid nitrogen and stored at −80°C. Subsequent treatment of the samples of the treatment group and the control group included extraction and purification of total RNA, construction of a cDNA library, transcriptome sequencing, and correlation analysis.

### Data Source

In this study, the Euphorbiaceae whole genome sequence, protein sequences, coding sequences and annotation files were obtained from the Oil Plant Database.^1^ The genes and protein sequences of the GST supergene family of Arabidopsis were obtained from the TAIR database (Araport11),^2^ *Jatropha curcas* from NCBI (RJC1_Hi-C),^3^ *Hevea brasiliensis* from NCBI (ASM165405v1, ASM1045892v1; see text footnote 3), and *Manihot esculenta* Crantz from Phytozome (Mesculenta_671_v8.0).^4^ The expression data of different tissues of *Arabidopsis thaliana* were obtained from Arabidopsis eFP Browser 2.0. The expression data of various tissues and biostresses of *R. communis* were obtained from the Gene Expression Omnibus (GEO) database (see text footnote 3), and the data for abiotic-stress-induced expression for *R. communis* treated with the herbicide sulfenesulfuron were obtained from previous experiments in our laboratory (Supplementary Table 7).

### *In silico* Identification of Members of the Glutathione S-Transferase Supergene Family in Euphorbiaceae

In order to identify the members of the GST supergene family in Euphorbiaceae, local BLASTP and HMMER comparisons were carried out (Mount, 2007; Finn et al., 2011). The protein sequences of Arabidopsis GST gene members were obtained from the TAIR database and used as query sequences for local BLASTP alignments. The hidden Markov models GSTN (PF02798) and GSTC (PF00043) of the typical GST domain were downloaded from the Pfam database for local HMMER alignment. The BLASTP results with threshold *E*-values ≤ 1e^–10^ and the HMMER results with threshold *E*-values ≤ 1e^–5^ were used as candidate sequences for the GST supergene family in Euphorbiaceae. The NCBI CDD^5^ and EBI InterPro^6^ tools were used to detect the domains of candidate sequences (Zdobnov and Apweiler, 2001; Lu et al., 2020), and the missing and incomplete sequences of the domains were manually deleted, in order to ensure the accuracy of the results. For the cases of multiple transcripts of the same gene, the first transcript was selected as the representative sequence, and finally the members of the Euphorbiaceae GST supergene family were identified. Clustal Omega was used for multiple sequence alignment (Sievers and Higgins, 2014), and Jalview was used to visualize the alignment results (Waterhouse et al., 2009). The physical and chemical properties of the identified members of the GST supergene family in Euphorbiaceae were analyzed using the ExPASy ProtParam tool^7^ (Gasteiger et al., 2005), and Plant-mPLoc was used for protein subcellular localization (Chou and Shen, 2007).

### Analysis of *Cis*-Acting Elements of the Glutathione S-Transferase Gene of Euphorbiaceae

Using genome and genome annotation files, the 1,500 bp upstream region of sequences of GST members were extracted by using a written script. PlantCare^8^ was used to predict the homeopathic elements of the GST gene in Euphorbiaceae (Lescot et al., 2002). The response element types were then manually summarized. GSDS 2.0 was used to visualize the predicted homeopathic elements (Hu et al., 2015).

### Analysis of the Glutathione S-Transferase Gene Structure and Conserved Protein Motif of Euphorbiaceae

Using the genome annotation file, the gene, intron and exon site information of the members of the GST supergene family were extracted by using the script, and the exon and intron lengths were calculated. MEME^9^ was used to analyze the conserved motifs of Euphorbiaceae GST proteins (Bailey and Elkan, 1994). The number of motifs was set to 15. The start and stop site information of the conserved motif was extracted from the script. The structures and conserved motifs of the GST supergene family in Euphorbiaceae were visualized using Evoview (Subramanian et al., 2019). A representative Euphorbiaceae GST protein was modeled using SWISS-MODEL (Waterhouse et al., 2018).

### Chromosome Mapping and Replication Analysis of the Glutathione S-Transferase Supergene Family in Euphorbiaceae

A distribution map of the GST gene on its chromosome was drawn using Circos software to confirm its position on the chromosome (Krzywinski et al., 2009). The replication pattern of the GST supergene family in Euphorbiaceae was analyzed by using MCScanX software^10^ (Wang et al., 2012). At the same time, collinear analysis was carried out between the Euphorbiaceae GST gene and Arabidopsis GST gene. Local Blast was used to build the database for the Euphorbiaceae and Arabidopsis protein sequence file. The protein sequence file was used as a query sequence for BLASTp alignment, and the *e*-value was set to 1 × e^–10^. The intraspecific and interspecific collinear relationships between Euphorbiaceae and *A. thaliana* were visualized using Circos. In Figure 4C. KaKs_Calculator2.0 was used to analyze the evolutionary selection pressure of replicating gene pairs, and the gene pairs that did not meet the threshold (*p*s > 0.75) were eliminated (Zhang et al., 2006); ggplot2 was used to visualize the results of the analysis of gene pairs that met the threshold (Ginestet, 2011).

### Analysis of Evolution and Development of the Glutathione S-Transferase Supergene Family

The multiple ClustalW sequences of *R. communis*, *J. curcas*, *H. brasiliensis*, and *M. esculenta* GSTs in Euphorbiaceae and *A. thaliana* were compared with the protein sequences of the model plant *A. thaliana*. An interspecific phylogenetic tree of Euphorbiaceae was constructed by deploying the adjacency method (NJ) using MEGAX software, and a phylogenetic tree of the Euphorbiaceae GST supergene family was constructed by deploying the maximum likelihood (ML) method with FastTree 2.1.11 software (Price et al., 2009; Kumar et al., 2018). The maximum likelihood method was deployed using the LG + G model. The adjacency method was deployed using the JTF model, with Bootstrap set to check 1,000 times and otherwise with default parameters, and using Evolview to visualize the evolutionary tree (Subramanian et al., 2019).

### Analysis of the Expression Patterns of the Glutathione S-Transferase Supergene Family in Euphorbiaceae

Abiotic stress data were obtained in previous experiments in our laboratory that included leaves, stems and roots of two varieties of *R. communis* treated with and without the herbicide sulfenesulfuron. Total RNA was extracted from each sample using a Plant Total RNA extraction Kit (Zomanbio, Beijing, China), according to the manufacturer’s instructions, and stored at −80°C. A total amount of 1 μg RNA per sample was used for the RNA sample preparations. The library was built with the Illumina NEBNext ^®^ Ultra™ RNA Library Prep Kit. The mRNA with polyA tail was enriched by Oligo (dT) magnetic beads, and then the mRNA was randomly interrupted by divalent cations in NEB fragmentation buffer. Using fragmented mRNA as a template and random oligonucleotides as primers, the first strand of cDNA was synthesized using the M-MuLV reverse transcriptase system, then the RNA chain was degraded by RNaseH, and the second strand of cDNA was synthesized from dNTPs in a DNA polymerase I system. The purified double-stranded cDNA was repaired by terminal repair, and a tail was added and sequenced. The cDNA of about 250–300 bp was screened by AMPure XP beads, PCR amplification was carried out, and the PCR product was purified by AMPure XP beads to yield the library. The library was initially quantified by a Qubit2.0 Fluorometer and diluted to 1.5 ng/μl, then the insert size of the library was measured with an Agilent 2100 bioanalyzer. When the insert size was satisfactory, qRT-PCR accurately quantified the effective concentration of the library (> 2 nM) to ensure its quality. After initial PCR amplification, the prepared libraries were constructed and sequenced on the Illumina NovaSeq 6000 System. Raw RNA-seq reads were preprocessed to remove the adaptor sequence, low-quality reads and contaminating sequences. Clean reads were then mapped onto the castor reference genome using Hisat2 2.2.1 (Kim et al., 2019). The gene expression levels were calculated using Htseq and count values were normalized to FPKM values using a script (Anders et al., 2014). Using DESeq2 for differential expression analysis, the genes consistent with |log_2_ (fold change) |>1 and padj < 0.05 were identified as differentially expressed genes (Love et al., 2014).

The expression data for various tissues of *A. thaliana* were obtained from Arabidopsis eFP Browser 2.0,^11^ with the tissues investigated including cotyledons, hypocotyls, first stem nodes, sepals, petals, stamens, carpels, mature pollen, seeds, leaves, flowers, roots, globular embryos, torpedo embryos and ovaries. The related expression data for various tissues of *R. communis* were obtained from the NCBI GEO database,^12^ with the tissues investigated including II/III endosperm, V/VI endosperm, germinated seeds, leaves, and male flowers (Brown et al., 2012). The expression data for *R. communis* leaves infected with *Sclerotinia sclerotiorum* were obtained from the NCBI GEO database (see text foot note 12). In order to improve the accuracy of the results, heat maps of gene expression were drawn using R-4.1.1 software^13^ and the Pheatmap package^14^ (Kolde, 2012), and the expression data were standardized.

### Analysis of Protein Interactions of the Glutathione S-Transferase Supergene Family in Euphorbiaceae

Interaction information for the GST supergene family proteins in Euphorbiaceae was searched by using the protein interaction database String (Szklarczyk et al., 2019). The results were imported into Cytoscape3.8.2 for interaction network visualization, and a color gradient from light to dark colors was used to show the Node Degree level of proteins in the network (Shannon et al., 2003).

## Data Availability Statement

The datasets presented in this study can be found in online repositories. The names of the repository/repositories and accession number(s) can be found in the article/Supplementary Material.

## Author Contributions

Y-SC, G-RL, and QD contributed to the conception and design of the study. QD performed the analysis and drafted the manuscript. Y-PQ, D-XY, and C-LZ contributed to the collection of plant materials. G-RL revised part of the manuscript. All authors contributed to manuscript revision, read, and approved the submitted version.

## Conflict of Interest

The authors declare that the research was conducted in the absence of any commercial or financial relationships that could be construed as a potential conflict of interest.

## Publisher’s Note

All claims expressed in this article are solely those of the authors and do not necessarily represent those of their affiliated organizations, or those of the publisher, the editors and the reviewers. Any product that may be evaluated in this article, or claim that may be made by its manufacturer, is not guaranteed or endorsed by the publisher.

## Footnotes

1
http://oilplants.iflora.cn

2
https://www.arabidopsis.org/

3
https://www.ncbi.nlm.nih.gov

4
https://phytozome-next.jgi.doe.gov

5
https://www.ncbi.nlm.nih.gov/cdd/

6
http://www.ebi.ac.uk/interpro/

7
https://web.expasy.org/protparam/

8
http://bioinformatics.psb.ugent.be/webtools/plantcare/html/

9
http://meme-suite.org/tools/meme

10
https://github.com/wyp1125/MCScanX

11
http://bar.utoronto.ca/efp2/Arabidopsis/Arabidopsis_eFPBrowser2.html

12
https://www.ncbi.nlm.nih.gov/geo/

13
https://www.r-project.org/

14
https://cran.r-project.org/web/packages/pheatmap/

## Supplementary Material

Supplementary Image 1 Results of cis-acting element analysis of GST supergene family in *Ricinus communis*.

Supplementary Image 2 Results of cis-acting element analysis of GST supergene family in *Jatropha curcas*.

Supplementary Image 3 Results of cis-acting element analysis of GST supergene family in *Manihot esculenta.*

Supplementary Image 4 Results of cis-acting element analysis of GST supergene family in *Hevea brasiliensis*.

Supplementary Image 5 Chromosome mappings, collinearity analyses, and evolutionary selection pressure analyses of GST genes in Euphorbiaceae. The purple lines represent the collinear relationship within the *A. thaliana* species, the red lines represent the collinear relationship within the *R. communis* species, the green lines represent the collinear relationship within the *M. esculenta* species, the black lines represent the collinear relationship within the *H. brasiliensis* species, the yellow lines represent the collinear relationship between *R. communis* and *M. esculenta* species, the blue lines represent the collinear relationship between *R. communis* and *A. thaliana* species, and the orange lines represent the collinear relationship between *M. esculenta* and *A. thaliana* species, the cyan lines represent the collinear relationship between *H. brasiliensis* and *M. esculenta* species, the brown lines represent the collinear relationship between *H. brasiliensis* and *R. communis* species, the dark green lines represent the collinear relationship between *H. brasiliensis* and *A. thaliana* species.

Supplementary Image 6 Results of qRT-PCR verification in *Ricinus communis*.

Supplementary Table 1 Basic information of the GST supergene family in Ricinus communis.

Supplementary Table 2 Basic information of the GST supergene family in *Jatropha curcas*.

Supplementary Table 3 Basic information of the GST supergene family in *Hevea brasiliensis*.

Supplementary Table 4 Basic information of the GST supergene family in *Manihot esculenta*.

Supplementary Table 5 Results of evolutionary selection stress analysis of GST supergene family in Euphorbiaceae.

Supplementary Table 6 The primers used in this study.

Supplementary Table 7 The links and accession numbers of the data in this study.
