# Supplementary figures and images for: Genome-Wide Identification, Evolution and Expression Analysis of the Glutathione S-Transferase Supergene Family in Euphorbiaceae
Source: Front Plant Sci. 2022 Mar 10;13:808279. doi: 10.3389/fpls.2022.808279 (PMC8963715; doi:10.3389/fpls.2022.808279)

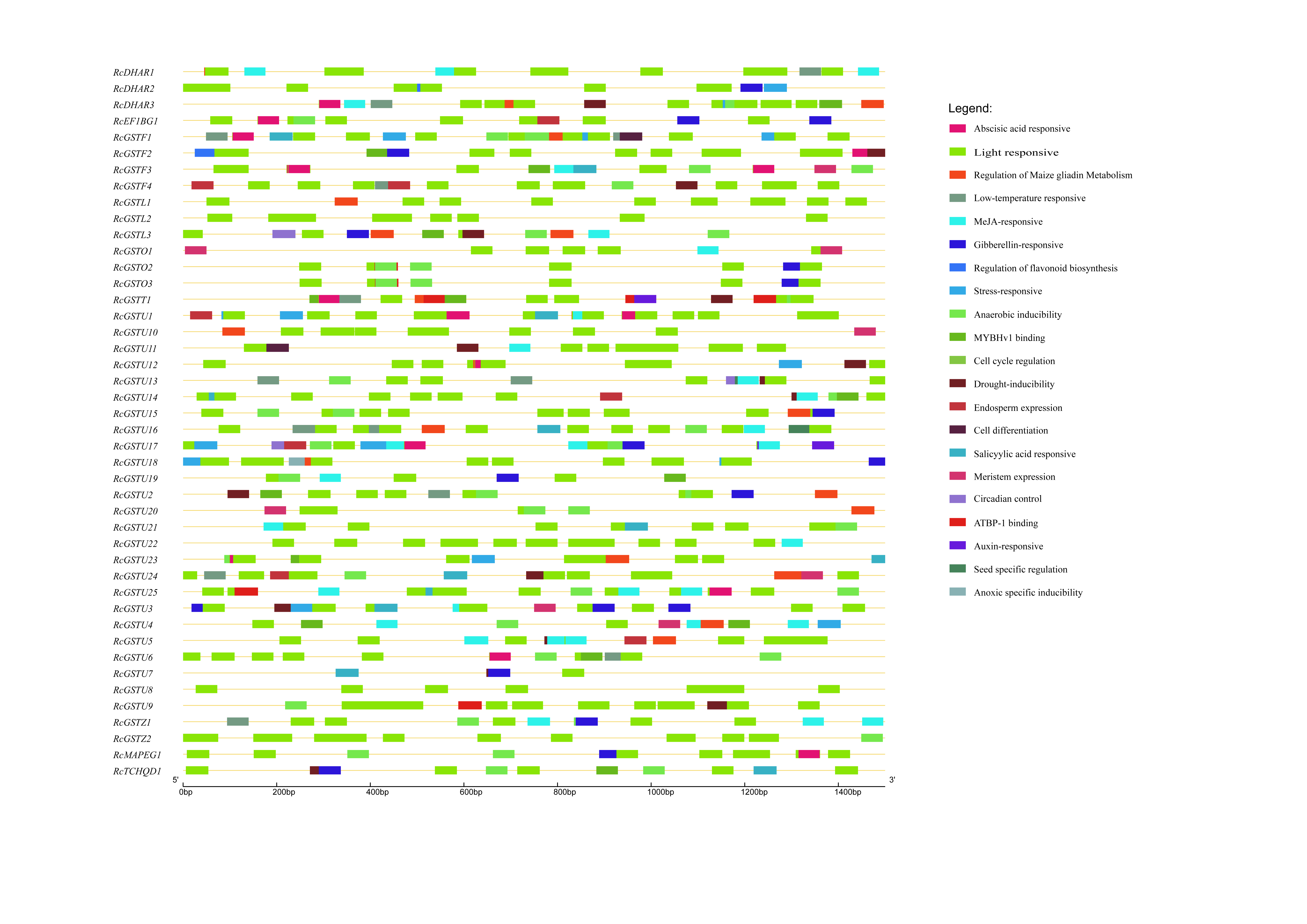

Supplement: Supplementary Image 1 — Results of cis-acting element analysis of GST supergene family in Ricinus communis. [file Image_1.JPEG]

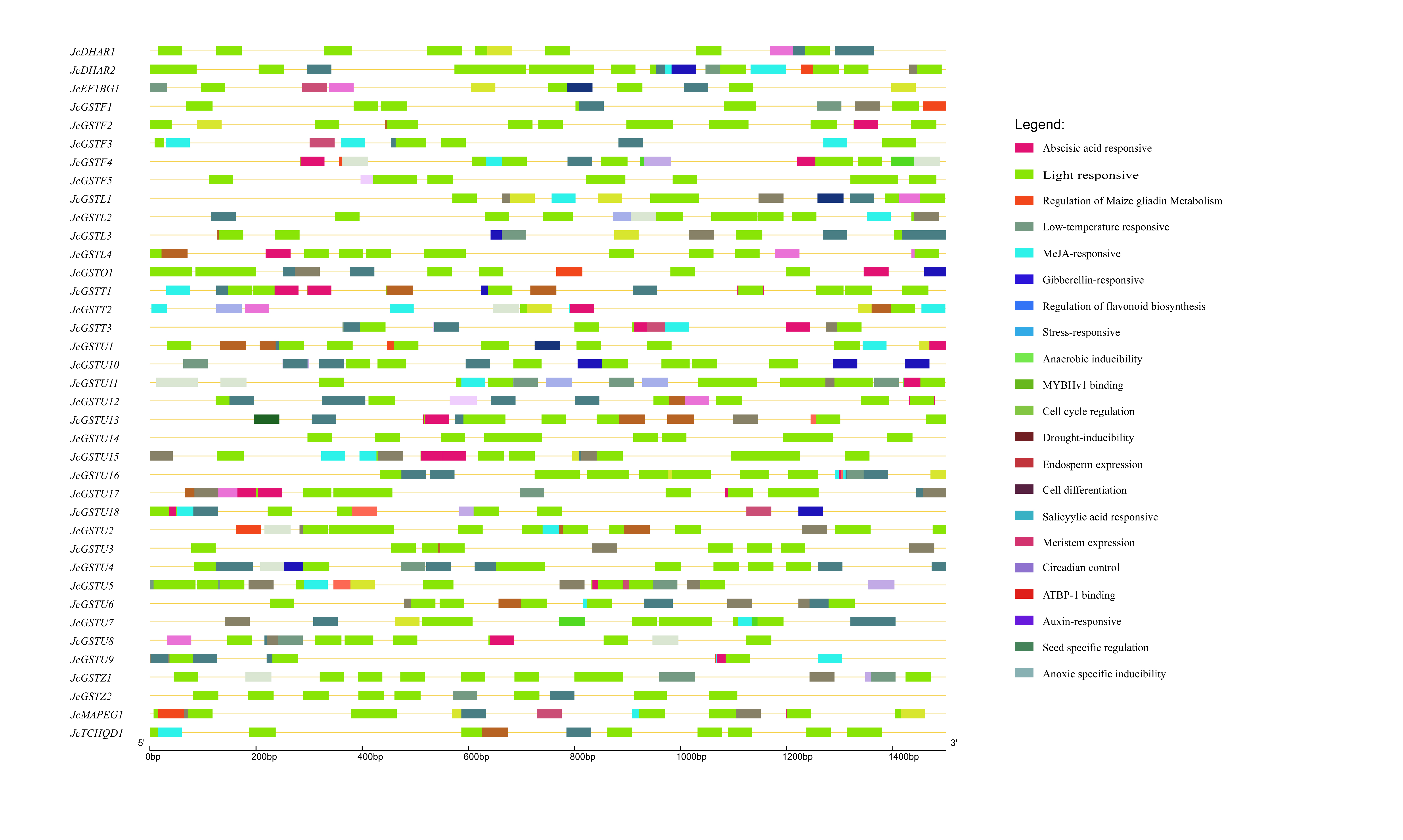

Supplement: Supplementary Image 2 — Results of cis-acting element analysis of GST supergene family in Jatropha curcas. [file Image_2.JPEG]

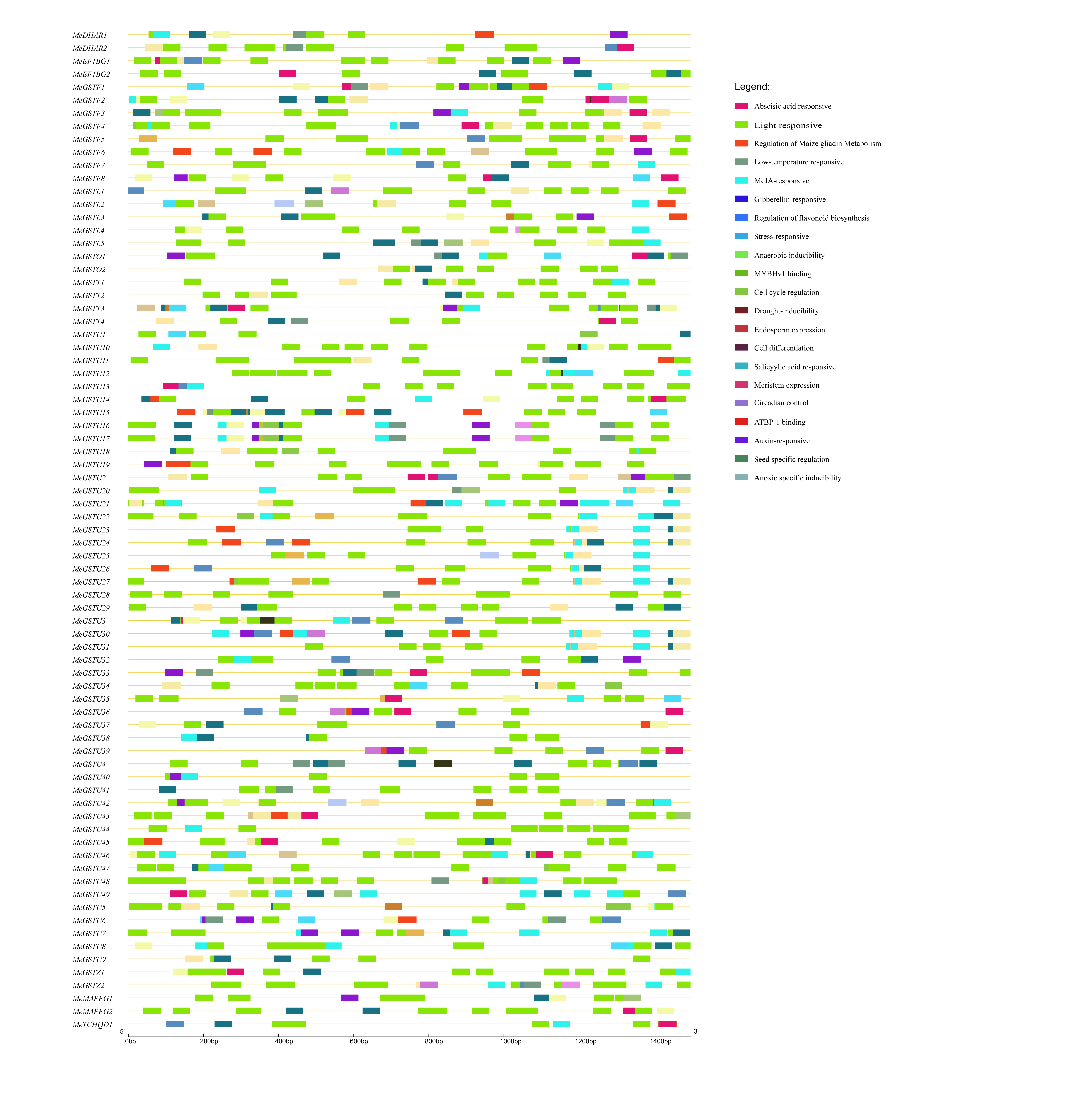

Supplement: Supplementary Image 3 — Results of cis-acting element analysis of GST supergene family in Manihot esculenta. [file Image_3.JPEG]

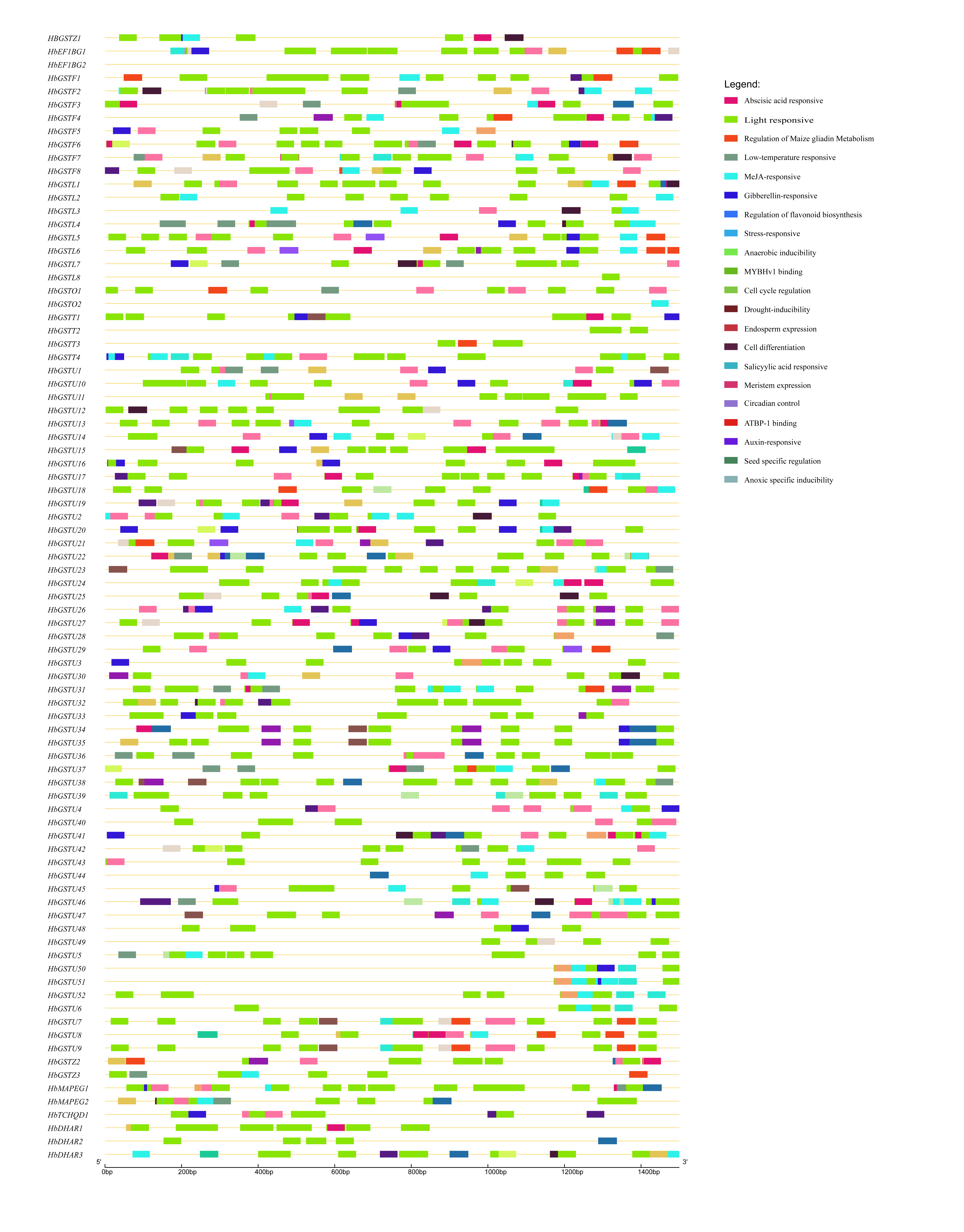

Supplement: Supplementary Image 4 — Results of cis-acting element analysis of GST supergene family in Hevea brasiliensis. [file Image_4.JPEG]

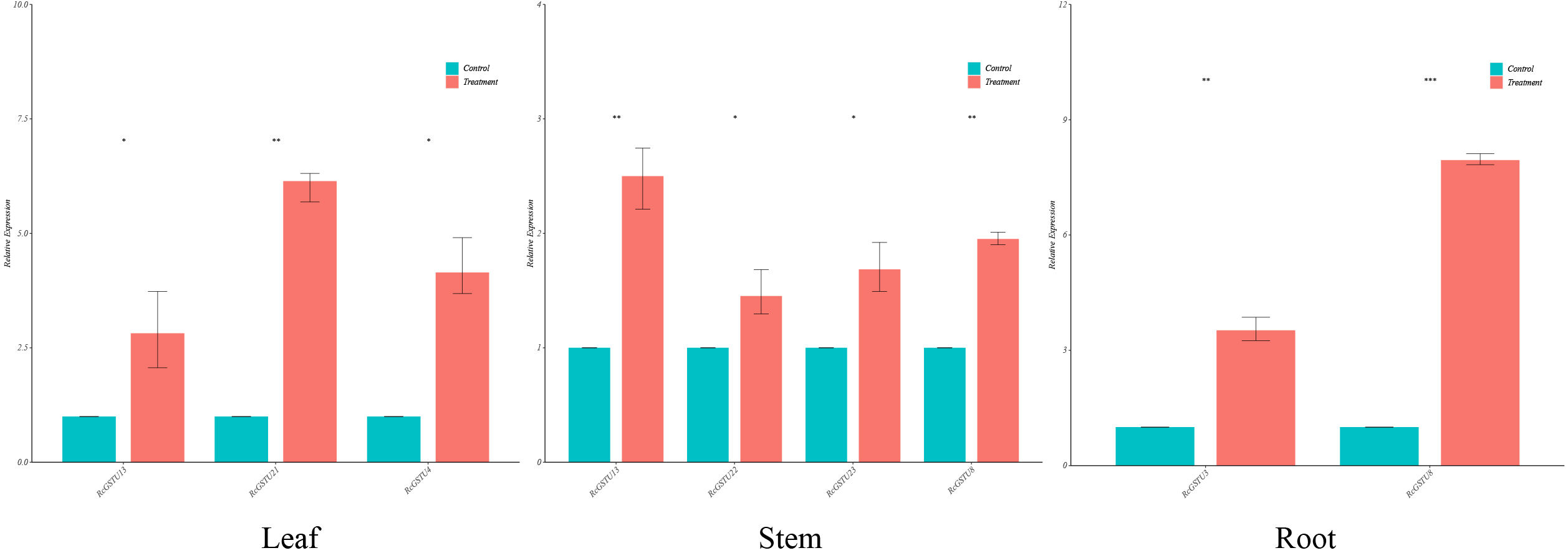

Supplement: Supplementary Image 6 — Results of qRT-PCR verification in Ricinus communis. [file Image_6.JPEG]
